# In-Capillary Photodeposition of Glyphosate-Containing Polyacrylamide Nanometer-Thick Films

**DOI:** 10.1021/acsapm.2c01461

**Published:** 2022-12-01

**Authors:** Jaroslaw Mazuryk, Katarzyna Klepacka, Joanna Piechowska, Jakub Kalecki, Ladislav Derzsi, Piotr Piotrowski, Piotr Paszke, Dorota A. Pawlak, Simone Berneschi, Wlodzimierz Kutner, Piyush Sindhu Sharma

**Affiliations:** † Electrode Processes Research Team, Institute of Physical Chemistry Polish Academy of Sciences, Kasprzaka 44/52, 01-224 Warsaw, Poland; ‡ Bio & Soft Matter, Institute of Condensed Matter and Nanosciences, Université catholique de Louvain, 1 Place Louis Pasteur, 1348 Louvain-la-Neuve, Belgium; § Functional Polymers Research Team, Institute of Physical Chemistry, Polish Academy of Sciences, Kasprzaka 44/52, 01-224 Warsaw, Poland; ∥ Microfluidics and Complex Fluids Research Team, Institute of Physical Chemistry Polish Academy of Sciences, Kasprzaka 44/52, 01-224 Warsaw, Poland; ⊥ Faculty of Chemistry, University of Warsaw, Pasteura 1, 02-093 Warsaw, Poland; # ENSEMBLE3 sp. z o. o., Wólczyńska 133, 01-919 Warsaw, Poland; ∇ Institute of Applied Physics “Nello Carrara”—National Research Council (IFAC-CNR), Via Madonna del Piano, 10, 50019 Sesto Fiorentino, FI, Italy; ○ Faculty of Mathematics and Natural Sciences. School of Sciences, Cardinal Stefan Wyszynski University in Warsaw, Wóycickiego 1/3, 01-938 Warsaw, Poland

**Keywords:** confocal micro-Raman spectroscopy, glyphosate, fused silica microcapillary, microfluidics, nanofilm, photopolymerization, polyacrylamide, silanization

## Abstract

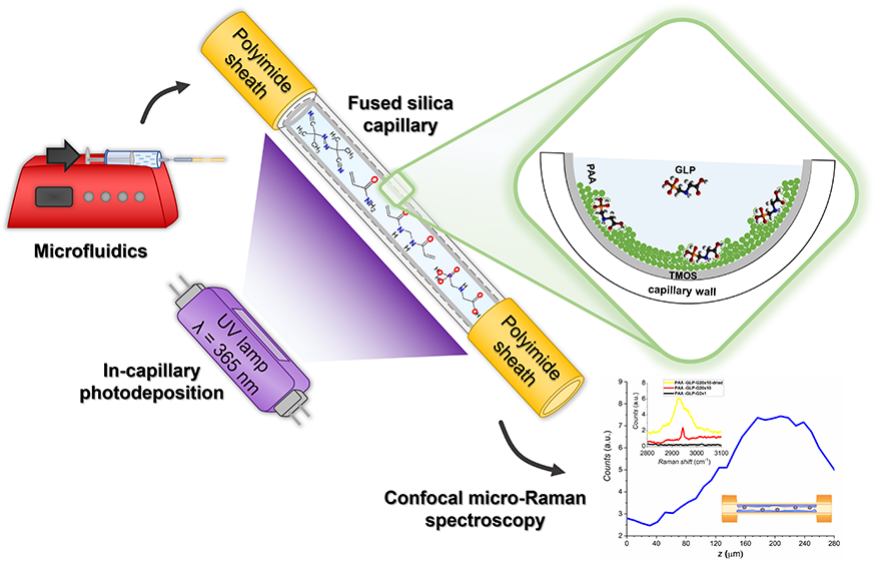

The present research reports on in-water, site-specific photodeposition of glyphosate (GLP)-containing polyacrylamide (PAA-GLP) nanometer-thick films (nanofilms) on an inner surface of fused silica (fused quartz) microcapillaries presilanized with trimethoxy(octen-7-yl)silane (TMOS). TMOS was chosen because of the vinyl group presence in its structure, enabling its participation in the (UV light)-activated free-radical polymerization (UV-FRP) after its immobilization on a fused silica surface. The photodeposition was conducted in an aqueous (H_2_O/ACN; 3:1, *v*/*v*) solution, using UV-FRP (λ = 365 nm) of the acrylamide (AA) functional monomer, the *N*,*N*′-methylenebis(acrylamide) (BAA) cross-linking monomer, GLP, and the azobisisobutyronitrile (AIBN) UV-FRP initiator. Acetonitrile (ACN) was used as the porogen and the solvent to dissolve monomers and GLP. Because of the micrometric diameters of microcapillaries, the silanization and photodeposition procedures were first optimized on fused silica slides. The introduction of TMOS, as well as the formation of PAA and PAA-GLP nanofilms, was determined using atomic force microscopy (AFM), scanning electron microscopy with energy-dispersive X-ray (SEM–EDX) spectroscopy, and confocal micro-Raman spectroscopy. Particularly, AFM and SEM–EDX measurements determined nanofilms’ thickness and GLP content, respectively, whereas in-depth confocal (micro-Raman spectroscopy)-assisted imaging of PAA- and PAA-GLP-coated microcapillary inner surfaces confirmed the successful photodeposition. Moreover, we examined the GLP impact on polymer gelation by monitoring hydration in a hydrogel and a dried powder PAA-GLP. Our study demonstrated the usefulness of the in-capillary micro-Raman spectroscopy imaging and in-depth profiling of GLP-encapsulated PAA nanofilms. In the future, our simple and inexpensive procedure will enable the fabrication of polymer-based microfluidic chemosensors or adsorptive-separating devices for GLP detection, determination, and degradation.

## 1 Introduction

Polyacrylamide (PAA) is the polymer of acrylamide (AA) monomers. In acrylamide polymerization, *N*,*N*′-methylenebis(acrylamide) (BAA) is typically used as the cross-linking monomer, thus incurring gel properties of the PAA. Depending on the degree of cross-linking and doping with both organic and inorganic materials, PAA can be an insoluble, colorless, transparent gel (hydrogel) or a linear, white, odorless, soluble powder. Contrary to AA, a neurotoxin relatively easily absorbed through the skin, PAA is nontoxic. Therefore, large-scale PAA production requires strict adherence to health and safety precautions.^1^ In the industry, PAA is produced by thermally activated free-radical polymerization (FRP) using ammonium persulfate and *N*,*N*,*N*′,*N*′-tetramethylethylenediamine as initiators.^2^

Recently, studies have delivered many examples of the photopolymerization of AA^3−11^ and copolymerization of AA with other monomers, such as acrylic acid^12^ and *N*-isopropylacrylamide.^13^ Moreover, photo-PAAs loaded with drugs, e.g., ibuprofen^14^ or thymol,^15^ have been fabricated. Regarding the application, PAA is a typical gel biomaterial used for medical diagnostics, relying on the electrophoresis of proteins and nucleic acids.^16^ Besides, it is helpful for cosmetics and food supplies as a stabilizer, thickener, and filler. Until recently, PAA has been used in plastic surgery to prepare soft tissues and artificial joint endoprostheses.^12^ Furthermore, the PAA hydrogel can successfully be exploited in pharmacy for the controlled release of drug sorbents^17^ and chemosensors.^18^

Herein, the PAA nanometer-thick films (nanofilms) were prepared by UV light (λ = 365 nm) irradiation of a prepolymerization aqueous (H_2_O/ACN; 3:1, *v*/*v*) solution of AA as the functional monomer, BAA as the cross-linking monomer, glyphosate (GLP) as the template, and azobisisobutyronitrile (AIBN) as the initiator of the (UV light)-activated free-radical polymerization (UV-FRP). UV-FRP was chosen because it is more versatile, enabling easy initiation and control of the polymerization compared to electropolymerization or thermopolymerization. Besides, UV-FRP allows for conferring spatially selective characteristics of the polymer growth, especially when the photopolymerization is combined with masking. Finally, a wavelength of 365 nm was chosen because many organic radicals, including AIBN, are photosensitive in this spectral range, thus may initiate UV-FRP efficiently.^19,20^

Over decades, GLP—a broad-spectrum systemic herbicide—has attracted the attention of scientists, agriculture workers, and public opinion because of its potentially harmful impact on human health. Being the most popular herbicide worldwide, GLP has been exploited as a template or analyte for various polymeric^21^ or artificial intelligence-based^22^ drug delivery systems and chemosensors operating in chromatographic,^21^ electrochemical,^23^ optical,^24^ immunochemical,^25^ microfluidic,^26^ or smartphone-assisted^27^ manners. Hence, this severe socioeconomic importance of GLP makes the present study considerable. Because GLP is photochemically and photophysically inactive, it cannot directly be determined using the most conventional optical methods. Similarly, only a limited number of studies concerned the impact of GLP on FRP^28,29^ and AA polymerizations.^30−32^ Because of the photochemical inertia of GLP, only its photopolymerizable derivatives, such as acrylated GLP, were used in UV-FRP.^33,34^ GLP or GLP-based herbicide intoxication-induced health issues, highlighted in clinical case reports, relate to hyperkalemia, oliguria, acidosis, digestive system malfunctions, cardiogenic shock, respiratory diseases, as well as neurological, hepatic, and kidney disorders.^35−38^ Hence, the construction of microfluidic nanomaterial-based adsorbents or sensors for rapid on-site GLP determination and degradation, especially in contaminated waters or body fluids, has become a challenge for analytical chemistry and materials science.

(UV-FRP)-assisted photodeposition on an inner surface of a fused silica capillary can easily be applied to, e.g., site-specific producing molecularly imprinted polymers (MIPs)^21,32^ and (GLP-MIP)-based adsorption or sensing systems.^32^ Accordingly, the method presented herein will be used for future photodeposition of PAA-GLP-MIPs on inner surfaces of hollow core whispering gallery mode (WGM) silica microresonators, such as microbubble and liquid core optical ring resonators, for devising microfluidic chemo- and biosensors with high performance and multiplexing features.^39,40^ The WGM resonators are miniaturized spherical or toroidal microlasers of excellent lasing properties applicable to in-air or microfluidic sensing.^39,40^ Recently, the optofluidic in-capillary WGM sensors were exploited in the photoactivated detection of antibodies,^39^ ultrasensitive lab-on-a-chip immunoassays of a vascular disease biomarker,^41^ and lasing in blood.^42^

The present study aimed to develop a method of GLP-binding PAA (PAA-GLP) deposition on the internal surface of a fused silica microcapillary with an inner diameter ID = 200 μm. A UV-FRP in an aqueous solution was employed to encapsulate GLP in a polymer network of AA and BAA, used as the functional and cross-linking monomers, respectively. Moreover, a procedure for the fused silica surface silanization with trimethoxy(octen-7-yl)silane (TMOS) was developed. TMOS was chosen arbitrarily because of the vinyl group presence in its structure, which enabled its participation in the UV-FRP after the immobilization on a fused silica surface.

Briefly, we first optimized the conditions of UV-FRP of AA and BAA in an aqueous solution in the presence of GLP. Next, we established a procedure for TMOS immobilizing on the surface of the fused silica slide and then on the inner surface of the fused silica microcapillary. Afterward, we encapsulated GLP in an AA–BAA polymer network on the TMOS-functionalized surfaces aided by UV-FRP mediation. With atomic force microscopy (AFM) and scanning electron microscopy with energy-dispersive X-ray (SEM–EDX) spectroscopy, we analyzed the properties of the nanofilms photodeposited on the fused silica slides. Finally, we examined the effectiveness of the photodeposition on the inner surface of the silica glass microcapillaries by micro-Raman spectroscopy. Thus far, in-depth micro-Raman spectroscopy has been used for sub-micro-structurization^43^ and vapor–liquid phase transition^44^ in a high-pressure microcapillary cell, as well as to study biological molecules and to identify primary human bronchial epithelial cells.^45^ Regarding GLP sensing, the GLP content was determined with surface-enhanced Raman scattering (SERS) spectroscopy using gold nanoparticles^46^ and silver composites.^47^ Our current research presents a successful application of micro-Raman spectroscopy to GLP-applied polymer science.

## 2 Materials and Methods

### 2.1 Materials

Acetonitrile (#271004, 99.8%, MW = 41.08 g/mol), acrylamide (#A9909, 99%, MW = 71.08 g/mol), azobis(2-methylpropionitrile) (#441090, 98%, MW = 164.31 g/mol), glyphosate (#45521, 98%, MW = 169.07 g/mol), *N*,*N*′-methylenebis(acrylamide) (#146072, 99%, MW = 154.17 g/mol), toluene (#244511, 99.8%, MW = 92.14 g/mol), and trimethoxy(7-octen-1-yl)silane (#452815, 80%, MW = 232.39 g/mol) were purchased from Merck (Poznan, Poland). Ethanol (#BA6480111, 99.8%, MW = 46.08 g/mol) and isopropanol (#BA1500111, 99.7%, MW = 60.11 g/mol) were from POCh (Warsaw, Poland). Hydrogen peroxide (#118851934, analytical grade, MW = 34.01 g/mol) and sulfuric acid (#115750013, 98%, MW = 98.08 g/mol) were procured from Chempur (Piekary Slaskie, Poland). Acetone (#50-8123, 99.8%, MW = 58.08 g/mol) was obtained from Linegal Chemicals (Warsaw, Poland). Fused silica slides were provided by TedPella (GE124, #26009; Redding, CA), while commercial fused silica microcapillaries were purchased from Postnova (#FSS-Z-200280; Landsberg, Germany).

### 2.2 Microfluidics

SyringePump NE-1000 infusion pumps were used in the microfluidics experiments. The technical parameters adopted include a maximum and minimum flow rate of 6.2083 and 0.0853 μL/min, respectively. All pumps were calibrated and adjusted to 5 mL syringes according to the manufacturer’s guidelines. The following flow rates in microcapillaries were used: 5 μL/min for the silanization with TMOS, then 15 μL/min for washing with anhydrous toluene, next 15 μL/min for washing with H_2_O, and finally 15 μL/min for the inner surface activation with the “piranha” solution (98% H_2_SO_4_: 30% H_2_O_2_; 7:3, *v*/*v*). For UV-FRP, the TMOS-functionalized microcapillaries were filled with respective prepolymerization solutions with a flow rate of 5 μL/min (see below). These conditions were preoptimized based on previous studies.^39^

### 2.3 Silanization

#### 2.3.1 Silanizing Fused Silica Slides

Fused silica slides with factory dimensions of 76.2 × 50.8 × 1 mm^3^ were cut into rectangular pieces with dimensions of 15 × 5 × 1 mm^3^ at the Institute of Electronic Materials Technology in Warsaw. Before silanization and UV-FRP, they were consecutively cleaned with isopropanol, ethanol, acetone, and water for 5 min in each solvent using an ultrasonic bath at room temperature, 20 (±1) °C. Then the slides were thoroughly dried in the air, and their surfaces were activated by slides immersing in the piranha solution for 1 h at 60 °C. After removing them from this solution, the slides were thoroughly rinsed with distilled water for 1 h. Finally, they were dried in an argon stream for 10 min.

Four cleaned fused silica slides were immersed in four vials for 5 and 16 h silanizations, each containing a 3 mL sample of 1 and 2 vol % anhydrous TMOS, respectively, dissolved in anhydrous toluene. The following four samples were prepared: “1%, 5 h”; “2%, 5 h”; “1%, 16 h”; and “2%, 16 h”. After silanizing, the slides were removed from the vials, rinsed with anhydrous toluene, then heated in an oven at 100 °C for 1 h to ensure the efficient condensation of TMOS methoxyl groups onto the silica surface,^48^ and finally dried in an argon stream for 10 min.

#### 2.3.2 Silanizing Silica Microcapillaries

Fused silica microcapillaries with an outer diameter and inner diameter of OD = 280 μm and ID = 200 μm, respectively, were used. On the outer surface, they have been coated with a 20-μm-thick polyimide sheath. It was demonstrated that for 25-μm-thick polyimide foils, the optical transmittance of electromagnetic radiation of wavelengths shorter than 400 nm is practically negligible.^49^ Hence, the polyimide sheath shielded the UV radiation of λ = 365 nm, thus masking it. Moreover, polyimide sheath’s fluorescence properties could appear during Raman spectroscopic–microscopic analysis and hamper it. Therefore, before silanization and UV-FRP, the outer polyimide sheath of the microcapillaries was burnt, and subsequently, the microcapillary was mechanically cleaned in organic solvents to remove combustion residues. As a result, a transparent window for UV-FRP was formed.

Then, the microcapillaries were washed by consecutive flushing with isopropanol, ethanol, acetone, and water for 5 min in each solvent at a flow rate of 15 μL/min, using a SyringePump NE-1000 infusion. Then, a “piranha” solution was infused at a flow rate of 15 μL/min for 30 min to activate their inner surfaces. Finally, the microcapillaries were washed with water at a flow rate of 15 μL/min for 6 min.

Afterward, the microcapillaries’ inner surfaces were silanized with the 2 vol % TMOS anhydrous toluene solution for 2 h at 5 μL/min. This concentration selection resulted from the preliminary optimization (fused silica slides; see above) and literature data.^39^ Next, the microcapillaries were rinsed with toluene (15 μL/min, 6 min) and then washed with water (15 μL/min, 6 min). Finally, the microcapillaries were heated at 100 °C for 1 h.^48^

### 2.4 (UV Light)-Activated Free-Radical Polymerization

#### 2.4.1 Optimizing Bulk UV-FRP in Aqueous Solutions

For all UV-FRPs, prepolymerization solutions of AA, BAA, AIBN, and GLP were prepared according to the data presented in Table 1. The reactants were first dissolved in a 3 mL sample of the H_2_O/ACN (3:1, *v*/*v*) solution in glass vials and then ultrasonicated for 30 min. ACN was used for two reasons, i.e., as a porogenic agent and to enhance the solubility of water-insoluble AIBN, a common free-radical generator initiating UV-FRP.^50^ In all cases, AIBN was added to the solution as the last component. Each solution was deoxygenated with an argon stream for 30 min. Herein, all UV-FRPs were conducted using the H_2_O/ACN mixed solvent solution because of the low solubility of GLP in organic solvents. Examples of successful in-water photopolymerizations have already been reported.^3,11^ Bulk UV-FRP was conducted by irradiating the solutions with a Herolab NU-4 4W UV lamp (λ = 365 nm) for 1 h. The vials filled with the test solutions were kept 5 cm away from the lamp to enable UV light transmission,^51^ on the one hand, and to avoid heating the samples, on the other.

**Table 1:** Prepolymerization (H_2_O/ACN; 3:1, *v*/*v*) Solutions for UV-FRP Performed on Fused Silica Slide Surfaces and Fused Silica Microcapillary Inner Surfaces^a^

		Content of prepolymerization solution components							
		AA		BAA		AIBN		GLP	
UV-FRP location	Sample name	*m* [mg]	*n* [mmol]	*m* [mg]	*n* [mmol]	*m* [mg]	*n* [mmol]	*m* [mg]	*n* [mmol]
In bulk	PAA×0.5	0.9	0.0125	2.3	0.0156	2	0.0122		
	PAA×0.5-GLP	0.9	0.0125	2.3	0.0156	2	0.0122	4	0.025
	PAA×1	1.8	0.025	4.8	0.03125	2	0.0122		
	PAA×1-GLP	1.8	0.025	4.8	0.03125	2	0.0122	4	0.025
	PAA×2	3.6	0.05	9.6	0.0625	2	0.0122		
	PAA×2-GLP	3.6	0.05	9.6	0.0625	2	0.0122	4	0.025
	PAA×5	9	0.125	24	0.156	2	0.0122		
	PAA×5-GLP	9	0.125	24	0.156	2	0.0122	4	0.025
	PAA×10	18	0.25	48	0.312	2	0.0122		
	PAA×10-GLP	18	0.25	48	0.312	2	0.0122	4	0.025
On-slide	PAA×5	9	0.125	24	0.156	2	0.0122		
	PAA×5-GLP	9	0.125	24	0.156	2	0.0122	4	0.025
In-capillary	PAA×1-Ar1	2.8	0.04	6.1	0.04	2	0.0122		
	PAA×1-Ar2	2.8	0.04	6.1	0.04	2	0.0122		
	PAA×10-Ar1	28	0.4	61	0.4	2	0.0122		
	PAA-GLP2×1	2.8	0.04	6.1	0.04	2	0.0122	2	0.01
	PAA-GLP8×1	2.8	0.04	6.1	0.04	2	0.0122	8	0.04
	PAA-GLP20× 1	2.8	0.04	6.1	0.04	2	0.0122	20	0.1
	PAA-GLP20×10	28	0.4	61	0.4	2	0.0122	20	0.1

a AA, acrylamide; BAA, *N*,*N*′-methylenebis(acrylamide); AIBN, azobisisobutyronitrile; GLP, glyphosate; *m*, mass; and *n*, number of moles.

#### 2.4.2 Optimizing On-(Fused Silica Slide) UV-FRP

To perform heat-free, site-specific, and controllable on-slide UV-FRP of AA, BAA, and GLP, the deoxygenated solution samples were dropwise dispensed on TMOS-functionalized fused silica slide surfaces and then UV light-irradiated in the same manner as described above (Table 1). During the irradiation, the slides with the drops were kept on the hand-made scaffold, horizontally mounted 5 cm above the UV lamp. The slides were irradiated from the bottom to avoid rapid evaporation and yield a homogeneous photoreaction of the AA and BAA monomers with the (slide surface)-confined TMOS molecules.

#### 2.4.3 Optimizing In-Microcapillary UV-FRP

In-microcapillary UV-FRP of AA, BAA, and GLP on TMOS-functionalized inner surfaces was conducted after the 15 min infusion at 5 μL/min of the deoxygenated prepolymerization solutions into microcapillaries. After infusion, the capillary terminals were immediately taped to avoid solution leakage while irradiating. The conditions of UV-FRP parameters included (i) a distance (1 and 5 cm) from the lamp, keeping the reaction time constant (20 min) and (ii) a duration of deoxygenation with argon, i.e., 30 min (“Ar1”) and 10 min (“Ar2”) (see Table 1). For micro-Raman imaging, the unbound photopolymer was flushed out from the microcapillary using water, and then the surface was imaged to analyze the on-surface-deposited polymers. The inner surfaces of the microcapillaries were rinsed twice with water at 5 μL/min for 6 min and then once at 15 μL/min for 6 min.

### 2.5 Atomic Force Microscopy

Atomic force microscopy (AFM) images were acquired on an AFM microscope coupled with a neaSNOM scattering-type scanning near-field optical microscope (attotube systems AG). Gold-coated silicon probes were used in the tapping mode. The AFM image data were acquired from the area of 10 × 10 μm^2^, 100 × 100 points. The images were baseline-corrected, if necessary.

### 2.6 Scanning Electron Microscopy with Energy-Dispersive X-ray Spectroscopy

Morphology and elemental analysis of the polymers were determined using a scanning electron microscope (FEI Nova NanoSEM 450) equipped with EDX and WDX detectors. For the SEM–EDX analysis, all of the polymer samples were drop-cast on the gold surfaces.

### 2.7 Confocal Micro-Raman Spectroscopy

Raman spectroscopy measurements were performed using a BRAVO Raman spectrometer (Bruker) equipped with a Duo LASER (700–1100 nm) laser (power < 100 mW). The spectra were acquired at a spectral resolution of 2–4 cm^–1^ and analyzed using OPUS ver. 2012 (Bruker Optic GmbH) software.

Confocal micro-Raman spectra were recorded at room temperature on a LabRAM HR Evolution spectrometer (Horiba Jobin Yvon) equipped with an Olympus BXFM-ILHS confocal microscope working in a backscattering geometry. A 532 nm Nd:YAG laser (Torus Laser, Laser Quantum, U.K.) of 1 mW power was used as an excitation source. Measurements were performed in a 50–4000 cm^–1^ spectral range, and the scattered signal was acquired with a 50× objective. The confocal hole diameter was 200 μm, and the diffraction grating was 1800 lines/mm. The spectra were collected with acquisition times varying from 3 to 15 s, with two accumulations each. For *z*-stack line mapping, an array of points along the *z*-axis within the depth of the capillary, spanning 280 μm with a step of 10 μm, was selected. The acquired micro-Raman spectra were baseline-corrected and smoothed if necessary.

Micro-Raman images of (photopolymer film)-coated and then flushed microcapillaries, and spectra of these photopolymers, were acquired in the *z*-axis (depth scan) in the 2800–3150 cm^–1^ range. This range was selected to analyze polymer characteristic bands assigned to the symmetric and asymmetric alkyl *ν*(CH_2_) vibrations. The following samples were analyzed: PAA×1, PAA×10, and their flushed counterparts (flushed PAA×1), as well as PAA-GLP2×1, PAA-GLP20×10, and their flushed counterparts. Micro-Raman *z*-stack imaging was performed with a 10 μm step, starting from the outer surface (position *z* = 0 μm), through the outer diameter and ending on the outer parallel surface (position *z* = 280 μm) to cover the full width of the microcapillary.

## 3 Results and Discussion

### 3.1 Silanization Optimizing

**Scheme 1:**
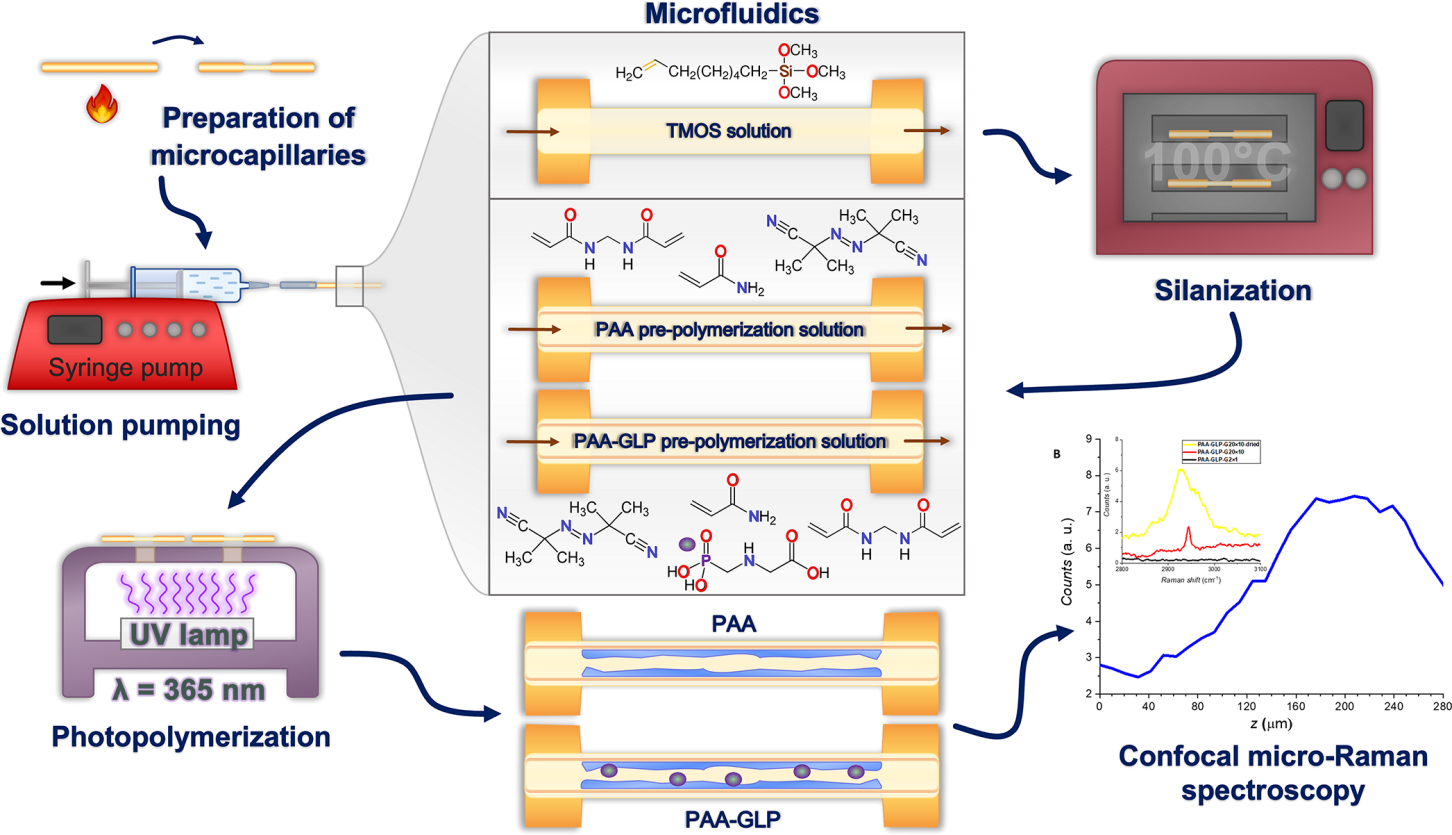
The Flowchart of (UV-FRP)-Assisted Photodeposition of PAA and PAA-GLP Films on a TMOS-Silanized Inner Surface of a Fused Silica Microcapillary

Our study aimed to develop a method for (UV-FRP)-assisted deposition of PAA and PAA-GLP on internal surfaces of fused silica microcapillaries (Scheme 1). Before UV-FRP, these surfaces were functionalized with TMOS. TMOS was selected because of its vinyl group capable of participating in UV-FRP, thus enabling the growing polymer chains anchoring to the fused silica surface. The silanization was optimized regarding the TMOS concentration (1 and 2 vol % in anhydrous toluene) and the silanization time (5 and 16 h). For this purpose, the cleaned slides were immersed in the following TMOS toluene solutions for the indicated time: 1%, 5 h; 1%, 16 h; 2%, 5 h; and 2%, 16 h. The efficiency of the TMOS deposition on the slides was examined by micro-Raman spectroscopy. For each slide, spectra were recorded at 2–3 selected spots.

#### 3.1.1 Optimizing the Silanization Time

**Figure 1:**
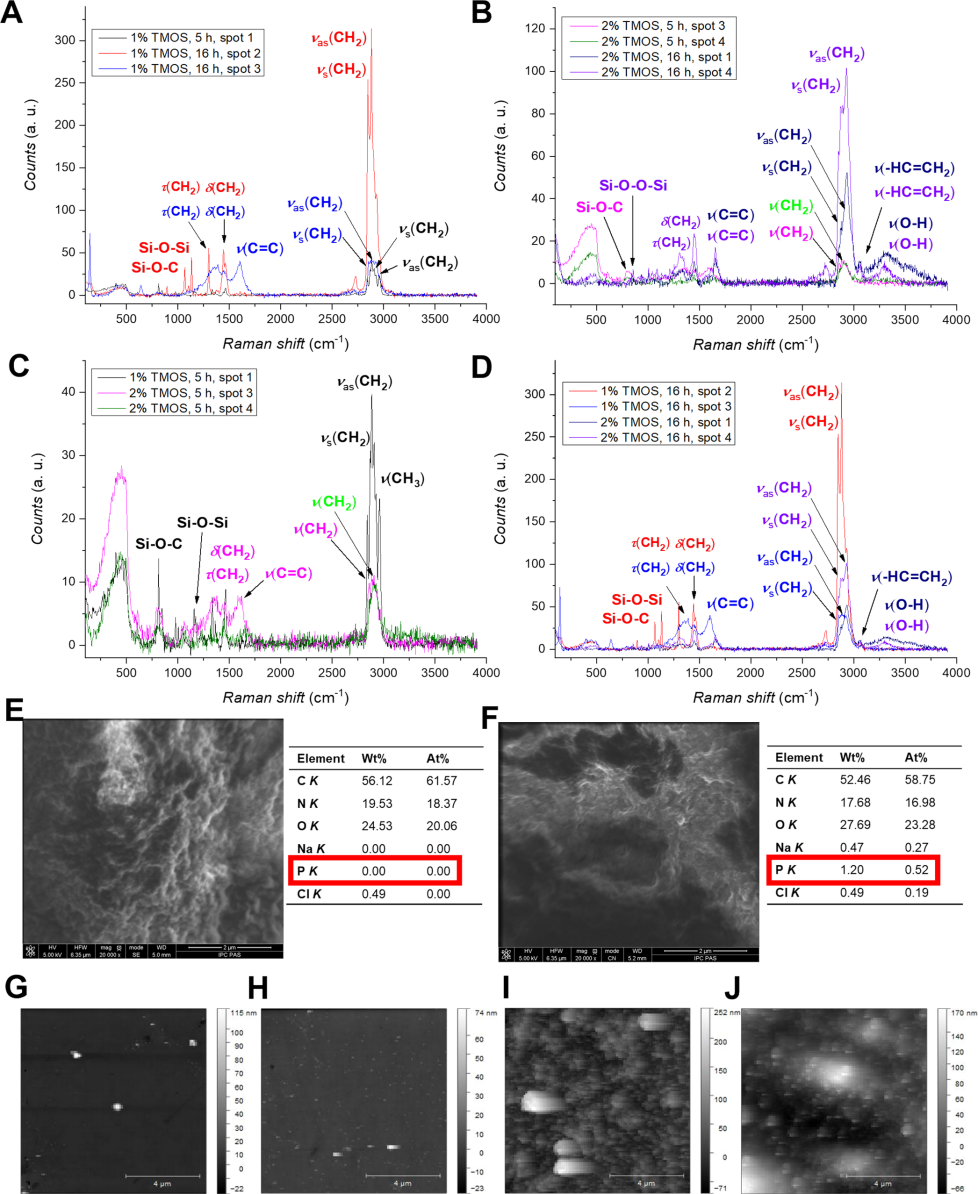
(A–D) Raman spectra of TMOS deposited on fused silica slides. Optimization of the silanization times (A) 5 and (B) 16 h and the TMOS concentration of (C) 1 and (D) 2 vol %. (E, F) SEM microimages and elemental composition of the (E) PAA and (F) PAA-GLP nanofilms drop-cast on gold plates. *K* – an electron shell. Scale bars on SEM images: 2 μm. (G–J) AFM images of (G) blank, (H) TMOS, (I) PAA, and (J) PAA-GLP. The PAA and PAA-GLP were deposited on TMOS-silanized fused silica slides. Scale bars on AFM images: 4 μm.

Figure 1A,B presents respective micro-Raman spectra of 1 and 2 vol % TMOS deposited on the fused silica slides after silanizing for 5 and 16 h. Anhydrous toluene was selected as the solvent for TMOS dissolving based on previous studies on the silanization of fused silica microcapillaries and microresonators.^39,52^ The spectra differ significantly, indicating ununiform silanization, presumably resulting from the immobilization of polysilanes of different structures. More importantly, the TMOS deposition differs depending on the time applied. Clearly, the 16 h silanization is more efficient in TMOS deposition (red and blue spectra in Figure 1A and violet and dark blue spectra in Figure 1B). In contrast, the 5 h silanization may result in inhomogeneous TMOS deposition, which is particularly visible for 1%, 5 h (black spectrum in Figure 1A) and 2%, 5 h (pink and green spectra in Figure 1B).

The efficient TMOS deposition is inferred from assignments of chemical groups to Raman band shifts as follows: (i) ∼1050 cm^–1^, vibrations of the Si–O–Si and Si–O–C bonds formed between the fused silica slide and TMOS; (ii) 1600 cm^–1^, vibrations of the double bond of the vinyl group; (iii) in the 2700–3000 cm^–1^ spectral range, vibrations of the C–H bonds of the TMOS alkyl chain; and (iv) 3100 cm^–1^, vibrations of the =C–H bonds of the TMOS vinyl group. These bands were present in the spectra of the samples of 1%, 16 h (spot 2) and 2%, 16 h (spots 1 and 4, respectively).

#### 3.1.2 Optimizing TMOS Concentration in Solutions for Silanization

The TMOS concentration effect on the silanization efficiency is presented in Figure 1 C (1 vol %) and Figure 1D (2 vol %). The most important conclusion from these results is the presence of the bands at ∼1600 cm^–1^ in the spectra of the 2%, 5 h (Figure 1C, pink spectrum) and 1%, 16 h (Figure 1D, blue spectrum) samples. These bands indicate the deposition of silanes with an active double bond. This inference is supported by the presence of a band at 3100 cm^–1^ assigned to the TMOS vinyl group (=C–H). This band is absent in samples deposited for 5 h. Moreover, in both spectra, there are bands in the ranges of 1200–1450 and 2700–3000 cm^–1^ attributed to the silane-specific CH_2_ group vibration and bands in the ranges of 500–1200 cm^–1^ characteristic of the Si–O–C bond vibration.

The Raman spectra are consistent with previous studies reporting the deposition of other silanes and polysilanes on quartz glass surfaces. For instance, this deposition method was used to perform the (UV light)-activated sol–gel phase transition of triethoxysilane and vinyl tetraethoxysilane.^53^ Similar results were obtained for the allylmethoxysilane sol–gel transition and an allyltrimethoxysilane monolayer self-assembly.^54^ Moreover, UV light irradiation was used to initiate the addition of mercaptopropyl trimethoxysilane to allyltrimethoxysilane and 7-octenyltrimethoxysilane.^55^ Finally, the usefulness of Raman spectroscopy was demonstrated in examining the ethylene and methacrylic acid co-polymer deposition on glass surfaces. These surfaces were preliminarily silanized with mixtures of methyltriethoxysilane with tetraethoxysilane, phenyltriethoxysilane with tetraethoxysilane, and methyltriethoxysilane with tetramethoxysilane.^56^

The efficiency of fused silica slide silanization with TMOS was confirmed by AFM imaging (Figure 1H). It indicates that the TMOS-modified surface did not much differ from a blank surface (Figure 1G). Based on this optimization and the data cited, for further UV-FRPs, we selected fused silica slides silanized with 2 vol % of TMOS for 16 h. Applying these optimized conditions ensured the successful deposition of TMOS with an active vinyl group. After PAA deposition, a clear difference in morphology was observed as a dense polymer structure was visible with occasional overgrowth (Figure 1I). AFM-assisted characterization of the PAA and PAA-GLP (Figure 1I,J, respectively) revealed homogeneous morphology and nanometer thickness of the polymers. Regarding the sizes, AFM imaging confirmed the nanometer thickness of PAA and PAA-GLP films, i.e., 262 and 179 nm, respectively. Figure 1J suggests low polymer growth in the presence of GLP, as the PAA-GLP grain density was lower than that of PAA. It may result from the GLP encapsulation or the hampering impact of GLP on the UV-FRP efficiency due to its interactions with AIBN or functional monomers while forming the prepolymerization complex. However, confirmation of these speculations requires further scrutiny.

#### 3.1.3 Silanizing Inner Surfaces of Fused Silica Microcapillaries

Optimizing the silanization of the fused silica slides aimed to deposit TMOS on the inner surfaces of the microcapillaries successfully. This step was monitored by Raman spectroscopy (Figure S1 in the Supporting Information). Clear bands at 1600–1700 and 2900 cm^–1^ assigned to vibrations of the C=C and CH_2_ bonds of TMOS, respectively, manifest the efficient TMOS in-capillary deposition. Moreover, the vibrations of the Si–O–Si and Si–O–C bonds in the 800–1100 cm^–1^ range suggest the interactions of TMOS with the silica substrate. However, the intensity of the bands assigned to vibrations of the C=C bond and the alkyl chain is relatively low compared to that of bands assigned to vibrations of the Si–O–Si and Si–O–C bonds. Apparently, the silanization efficiency was low, presumably because of too short silanization time or insufficient fused silica surface activation during the “piranha” cleaning.

Our conclusions are consistent with those of other studies on microcapillary silanization. For example, chloromethylsilane was used to functionalize microcapillaries and detect the ofloxacin antibiotic using liquid–liquid interface systems.^57^ In another study, vinyl 3-(trimethoxysilyl)propyl methacrylate served as both the silanizing agent and functional monomer for 1,6-butanediol methylacrylate photopolymerization and deposition of the resulting film on surfaces of fused silica microcapillaries.^52^ In turn, mixtures of 3-(trimethoxysilyl)propyl methacrylate and 3-(4-(methacryloxysilyl)propyl)trimethoxysilane were used to silanize capillaries with tri-*n*-butylborane surfaces.^58^ Ultimately, the quartz, silicon, and mica surfaces silanizing with triethoxysilane helped to deposit photoinduced molecular motors thereon.^59^

### 3.2 Photopolymerizing AA–BAA-GLP

#### 3.2.1 Photopolymerizing AA–BAA-GLP in Aqueous Solutions

**Figure 2:**
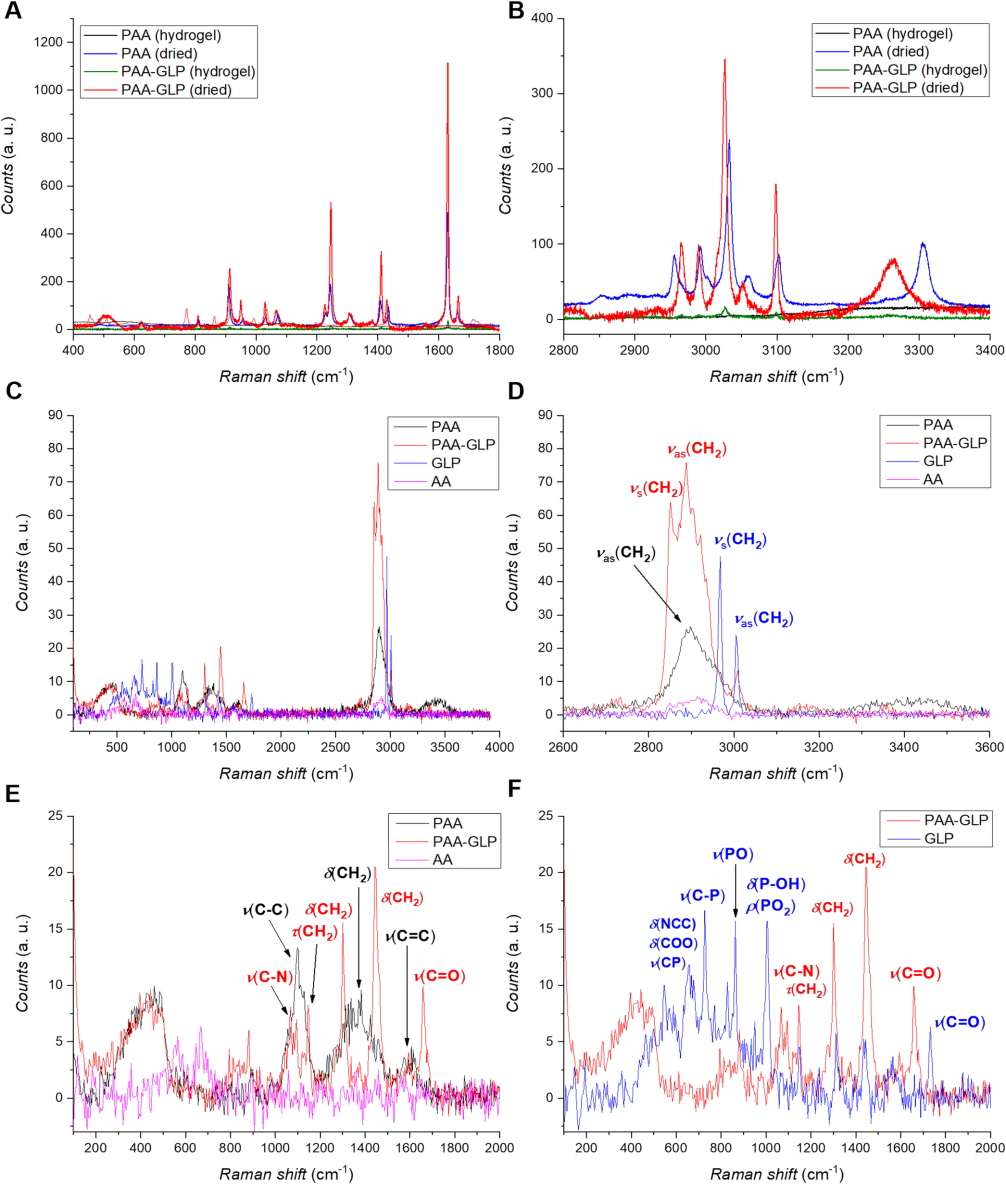
(A, B) Raman spectra for PAA and PAA-GLP hydrogels and dried gel samples in the (A) 400–1800 and (B) 2800–3400 cm^–1^ ranges. (C–F) Raman spectra for AA, GLP (drop-cast), as well as PAA and PAA-GLP immobilized on TMOS-functionalized fused silica slides in the (C) 400–4000, (D) 2600–3600, and (E, F) 100–2000 cm^–1^ ranges.

Hydrogels, as well as air-dried PAA and PAA-GLP, were examined using SEM–EDX (Figures 1E,F and S2 in the Supporting Information) and Raman spectroscopy (Figure 2A,B). The SEM microphotographs show clear polymer structures characterized by porosity incurred by the ACN porogen in UV-FRP.^60−62^ Moreover, despite similar PAA and PAA-GLP nanofilm thickness, GLP slightly impacted polymer roughness and porosity. Presumably, these features may facilitate GLP encapsulation. This speculation is supported by the EDX spectroscopy results of measurements for PAA-GLP samples drop-cast on gold plates. These results convincingly indicate the presence of GLP-specific phosphorus atoms in the PAA-GLP matrix (1.2 wt %, 0.52 atom %; Figure 1F). The recorded Raman spectra confirmed the GLP effect on the PAA-GLP structure. Figure 2C–F shows Raman spectra of the hydrogel and dried samples of PAA and PAA-GLP. In the 400–1800 cm^–1^ range, the PAA and PAA-GLP spectra do not differ significantly from each other. We explain the absence of GLP-characteristic bands in the PAA-GLP spectrum (e.g., PO at 1000 cm^–1^; see Figure 2F) by very low GLP content in the PAA-GLP, as mentioned above with respect to the EDX spectra (Figures 1E,F and S2 in the Supporting Information). In contrast, in the 2800–3400 cm^–1^ range, the most remarkable difference concerns the intensities of bands for dried samples, which are significantly higher than those for hydrated samples (Figure 2B, black and green spectra versus blue and red spectra). By assigning Raman bands in the 2800–3200 cm^–1^ range to hydroxyl *ν*(O–H) and amino *ν*(N–H) group vibrations, we explain this phenomenon by the presence of water entrapped in hydrogels. This water absorbs the incident light, thus decreasing the output signal intensity. Besides, GLP presumably interacts with the polymer’s N–H bond, manifested by the band at 3200–3350 cm^–1^, apparently GLP’s most significant impact on PAA (Figure 2B). Furthermore, concerning the difference between the spectra of dried PAA and PAA-GLP samples, the band evidently shifts in the latter (Figure 2B). This shift indicates the GLP binding by PAA, possibly through the hydrogen bonding between the PAA amide group and the GLP carboxyl and/or phosphonate group. Moreover, the GLP-induced spectral changes are in the 2590–3000 cm^–1^ range. These changes are assigned to symmetric and asymmetric *ν*(CH_2_) vibrations, thus indicating the influence of GLP on the structure of the polymer chain itself. Worth mentioning, there are two bands, one at 1600 cm^–1^ (Figure 2E) and the other in the 3000–3100 cm^–1^ range (Figure 2D), assigned to *ν*(C=C) and *ν*(=C–H) vibrations of AA and BAA monomers, respectively. They demonstrate the presence of nonpolymerized monomers, apart from the PAA and PAA-GLP.

#### 3.2.2 Photopolymerizing AA–BAA-GLP on Fused Silica Slides

UV-FRP on fused silica slides was performed by irradiating the prepolymerization solutions dropwise dispensed on TMOS-functionalized slides. The samples were irradiated for 10 min (λ = 365 nm) using a UV lamp placed 5 cm underneath the slides. After gentle washing away of the residual unreacted substrates and unbound polymers with water, the on-surface polymers were analyzed by AFM (Figure 1G–J) and Raman spectroscopy (Figure 2C–F). AFM images present results of both the 16 h TMOS deposition from 2 vol % TMOS in anhydrous toluene on fused silica slides and the UV-FRP of the H_2_O/ACN (3:1, *v*/*v*) solution of AA, BAA, and GLP. As shown, TMOS forms polysilanes’ islands rather than monolayers, presumably resulting from the prolonged deposition of TMOS applied at a relatively high concentration. However, as evidenced by Raman spectra (Figure 1A–D), these conditions enable depositing TMOS with an intact vinyl group that is required to copolymerize with AA and BAA, thus anchoring the photopolymers to the fused silica surface. By analyzing AFM images of PAA and PAA-GLP, we conclude that PAA forms a thicker (252 nm thick) and denser film than the PAA-GLP film (170 nm thick). Hence, GLP, present in the prepolymerization solution, decreases the efficacy of on-surface UV-FRP of AA and BAA. Most likely, GLP acts as an inhibitor of UV-FRP by deactivating free radicals of AIBN, as presented in previous studies on GLP-mediated FRP,^28,29^ including FRP of AA.^30−32^

Although our inference on the GLP role in UV-FRP seeks further scrutiny, it seems to be supported by the Raman spectra recorded (Figure 2C–F). Apparently, the PAA and PAA-GLP spectra differ significantly. For PAA-GLP, in the 2800–3100 cm^–1^ range, bands assigned to the GLP *ν*(CH_2_) vibrations (Figure 2D, blue spectrum) are shifted and superimposed; thus, the PAA-GLP band (red spectrum) is more intense than this band for PAA (black spectrum). This result confirms GLP’s significant influence on the AA–BAA UV-FRP or hydrogel formation, as presented in Figure 2A,B. However, these changes in the *ν*(CH_2_) vibrations of PAA and PAA-GLP may also result from different contributions of the methylene groups of GLP, AA, and polymers. Further analysis would be required in this regard. Continuously, the absence of bands assigned to the H_2_C=CH– moiety vibrations (expected at ∼3100 cm^–1^) suggests efficient UV-FRP of AA and BAA.^63−65^ In turn, in the range of 1000–1800 cm^–1^ (Figure 2E,F), a characteristic GLP stretching *ν*(CP) and *ν*(PO) vibration bands at ∼700–900 cm^–1^ as well as bending *δ*(P-OH) and rocking *ρ*(PO_2_) vibration bands at ∼1000–1050 cm^–1^ are present in the GLP spectrum but are absent in the PAA-GLP spectrum. Possibly, this band shifts, affecting the band of the C–N bond vibration in PAA (Figure 2F, red spectrum). Regarding the absence of GLP’s characteristic (PO_2_) group-assigned signal at ∼1000 cm^–1^, we speculate that this group is prone to forming complexes and/or hydrogen bonding during GLP interaction. For example, the PO_2_ group interactions were found in metal–ligand GLP–Cu^2+^ complexes.^66^ Expectedly, during UV-FRP, GLP’s phosphonate and carboxyl groups form hydrogen bonds with amine and/or amide groups of PAA. PAA would encapsulate GLP in the polymer network through these interactions, thus forming PAA-GLP. It is also possible that this interaction occurs at the stage of formation of the prepolymerization complex between GLP and functional monomers of AA. However, these speculations require further studies. Similarly, in the 1000–1200 cm^–1^ range, bands assigned to the polymer *ν*(C–C) vibrations in the PAA-GLP spectrum are less intense than in the PAA spectrum. In contrast, PAA-GLP C–N and methylene group’s C–H vibrations are more intense than those of PAA. Most likely, these differences indicate conformational changes resulting from upon PAA binding (encapsulation) of GLP.^63−65^ Regarding the 1200–1500 cm^–1^ range, generally populated by bands assigned to the twisting *τ*(CH_2_) (1200–1300 cm^–1^) and bending *δ*(CH_2_) (1300–1500 cm^–1^) vibrations, the GLP-characteristic CH_2_ group-assigned bands overlap corresponding bands in the PAA-GLP spectrum (Figure 2F), confirming a significant influence of GLP on the structural properties of PAA-GLP. Furthermore, in the 1500–1800 cm^–1^ range, both PAA and PAA-GLP spectra reveal bands assigned to *ν*(C=C) vibrations of free vinyl groups of AA or TMOS that avoided successful UV-FRP (Figure 2E). However, only PAA-GLP shows the band characteristic of the carbonyl group (C=O), also observed in the spectrum for GLP, albeit shifted (Figure 2F). This shift may result from the GLP carboxyl group interaction with PAA, possibly suggesting the GLP binding by PAA, as reported elsewhere.^30,31,63−65^

#### 3.2.3 AA–BAA-GLP Photopolymerizing on Inner Surfaces of Microcapillaries

To date, the PAA deposition on the inner surfaces of microcapillaries has mainly been used to construct capillary electrophoresis devices for routine separation of peptides and proteins, as well as nucleosides, nucleotides, and nucleic acids. Recently, PAA film-coated fused silica capillary columns have been used for chromatographic determination of a nucleoside. To this end, the PAA film was deposited by a 10 min free-radical photopolymerization of acrylamide.^67,68^

**Figure 3:**
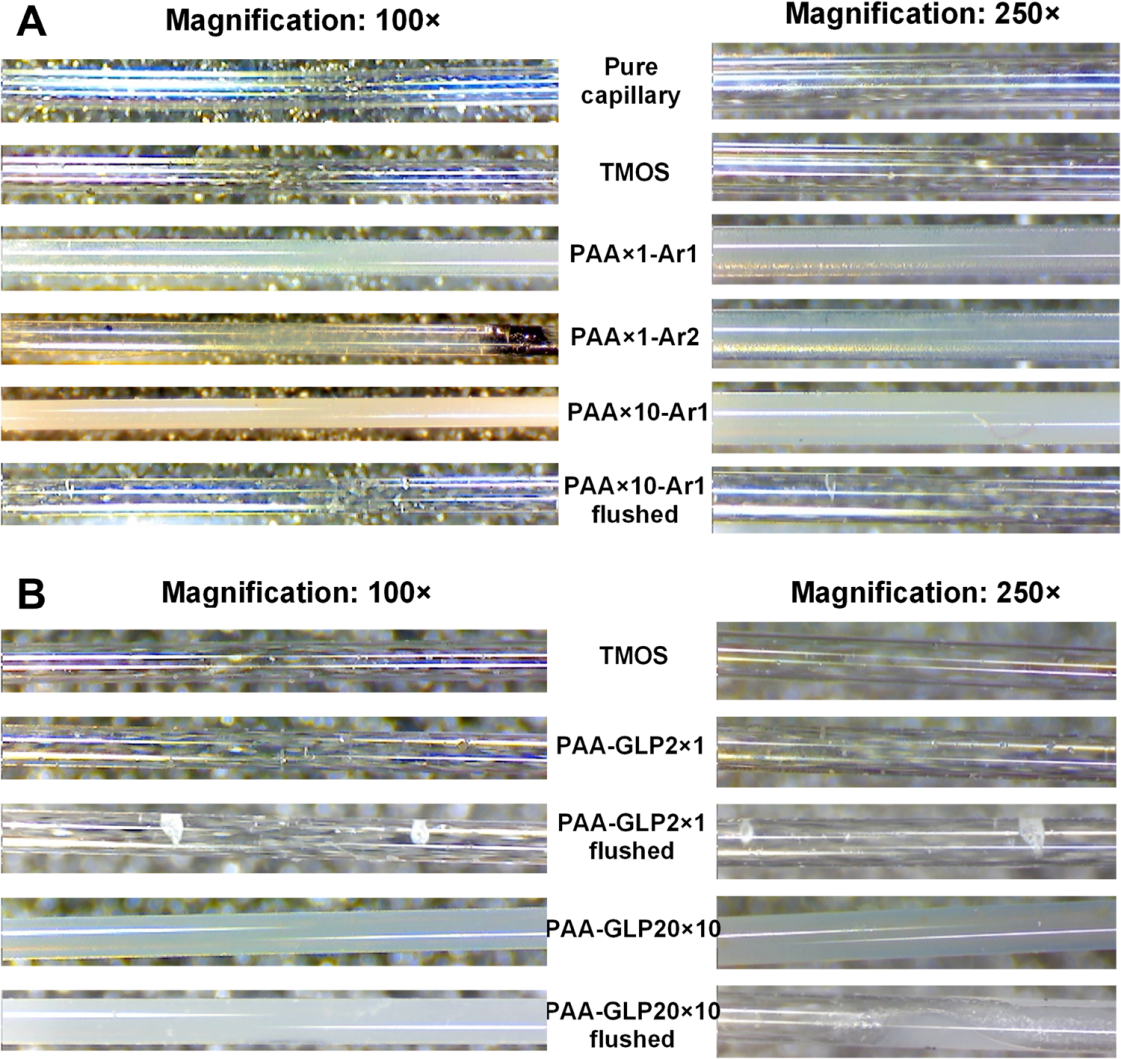
Images (magnifications 100× and 250×) of fused silica microcapillaries functionalized with TMOS, (A) PAA and (B) PAA-GLP. Ar1 and Ar2, samples of the prepolymerization solution deoxygenated in an argon stream for 30 and 10 min, respectively. AA1 and AA10, concentrations of AA; GLP2 and GLP20, concentrations of GLP.

Our study aimed to establish a procedure for depositing GLP-containing PAA (PAA-GLP) on the inner surfaces of microcapillaries using site-specific UV-FRP (Scheme 1). It was motivated by the future designing and fabricating of a microfluidic PAA-based adsorbent and/or chemosensor for GLP. For that, the inner surfaces were presilanized with 2 vol % TMOS for 2 h (Figure S1 in the Supporting Information), then filled in with the prepolymerization solutions (containing AA, BAA, GLP, and AIBN), and then UV light-irradiated from a distance of 1 cm for 5 min. These silanization conditions were preliminarily optimized. Figure 3A,B shows the respective microphotographs of capillaries internally coated with PAA and PAA-GLP in AA and GLP dose-dependent manners.

Concerning the TMOS layer on the microcapillary inner surface, no apparent difference between the uncoated (blank) and the TMOS-coated microcapillary surfaces is perceptible at the applied magnifications of 100× and 250×. However, successful silanization was confirmed by confocal Raman spectroscopy spectra (Figure S1 in the Supporting Information). Furthermore, microphotographs demonstrate the adequate argon purification (deoxygenation) impact on the prepolymerization solutions (Figure 3A). Apparently, longer deoxygenation (PAA×1-Ar1, 30 min) results in more efficient and controllable in-capillary UV-FRP, manifested by the deposition of the PAA film denser than the PAA×1-Ar2 film (10 min deoxygenation).

**Figure 4:**
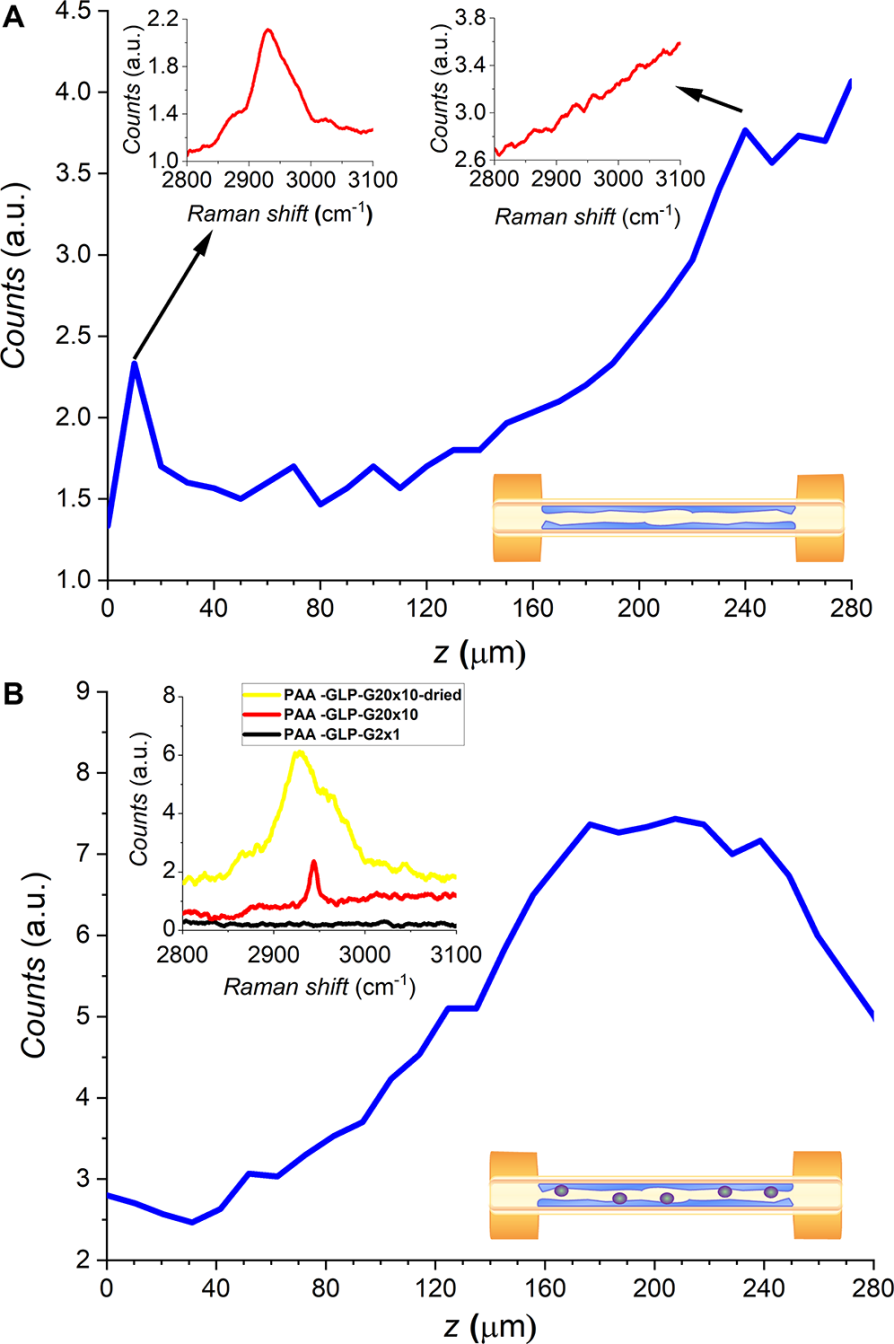
Micro-Raman line scan along the *z*-axis (in-depth scan) of (A) PAA×1 and (B) PAA-GLP20×10 in the flushed fused silica microcapillaries. Mapping was performed in the *z* = 0–280 μm range; integrated Raman intensity in the 2850–3050 cm^–1^ range [*ν*(C–H)] was plotted. Respective insets represent the Raman spectra in the 2800–3100 cm^–1^ range. PAA×1 and PAA-GLP-G20×10 (red spectra) were used for the micro-Raman *z*-line scan analysis.

Concerning dose-dependent effects, using 10-fold more concentrated AA and BAA at a constant AIBN concentration (PAA×10-Ar1) clearly results in more efficient UV-FRP. Unbound PAA was easily flushed out from the capillary, indicating the successful optimization of UV-FRP AA at the preceding stages. The (micro-Raman spectroscopy)-based analysis of the PAA-coated microcapillary inner surfaces is presented (Figure 4A) and discussed below.

Regarding PAA-GLP, the images visualize (Figure 3B) the dose-dependent impact of GLP on the in-capillary UV-FRP efficiency. A brief comparison of respective PAA and PAA-GLP images reveals that the addition of GLP to the prepolymerization solution at the GLP:AIBN molar ratio of 1:1 decreases the UV-FRP yield (Figure 3B; PAA-GLP2×1), while the addition of GLP in 10-fold excess (Figure 3B; PAA-GLP20×10) insignificantly changes the UV-FRP, indicating the encapsulation of GLP by the PAA matrix. Furthermore, by comparing polymer contents (thicknesses) in PAA×1 and PAA-GLP2×1 as well as PAA×10 and PAA-GLP20×10, one may infer that GLP hinders UV-FRP because of significantly lower polymer contents in the respective GLP-containing samples; similar behavior was confirmed by AFM imaging (Figure 1J). However, this inference requires confirmation by microanalysis of on-surface-deposited PAA-GLP using more sensitive analytical tools (Figure 4B). To this end, the unbound PAA-GLP was effectively flushed out from the microcapillary.

#### 3.2.4 Confocal Micro-Raman Spectroscopy

The efficacy of the photoassisted in-capillary deposition of the PAA and PAA-GLP films was evaluated by confocal micro-Raman spectroscopy (Figure 4A,B, respectively). The inset in Figure 4A presents micro-Raman spectra of PAA×1 photodeposited on the inner surface of the microcapillary. In all cases, measurements were carried out for the 2800–3150 cm^–1^ range, and the integrated intensity of the C–H vibration band in the 2850–3050 cm^–1^ range was plotted in the scan. The surface unbound photopolymer was flushed out from the microcapillary before microimaging. As shown, band intensities are relatively low because the silanization resulted in forming a thin adsorbate layer. The strongest signal was acquired from *z* = 0–40 μm, as presented in the respective inset. The Raman signal gradually disappeared for measurements at greater depths (*z* > 40 μm). An increase in intensity for *z* > 150 μm is caused by a higher background (the Raman spectrum collected for *z* = 240 μm is shown in the corresponding inset), most likely originating from general scattering and signal losses while passing through the capillary. Further studies are necessary to dispel these inconsistencies. Nevertheless, this result confirms the effectiveness of the PAA photodeposition on the inner surface of the microcapillary.

A microcapillary internally coated with the PAA-GLP20×10 film was similarly mapped with micro-Raman spectroscopy. Raman spectra were recorded for the hydrogel (wet) and flushed (dried) PAA-GLP at two concentrations (Figure 4B, inset). The intensity was the highest for the flushed/dried PAA-GLP20×10 (yellow spectrum in the inset). The C–H vibration band consisted of two components, as opposed to a one-component band for the wet/hydrogel PAA-GLP20×10 sample (Figure 4B, red spectrum). However, the yellow spectrum was recorded for a block of the photopolymer deposited on the surface (Figure 4B, inset). Because of the inhomogeneous coverage of the dry microcapillary, it was disregarded for the analysis. The wet/hydrogel PAA-GLP20×10 microcapillary spectrum presents a moderate intensity band in the 2800–2900 cm^–1^ range, assigned to polymer *ν*(C-H) vibrations; this system was then analyzed using micro-Raman spectroscopy. The latter analysis confirmed the presence of PAA-GLP20×10 on the inner bottom surface of the microcapillary (a gradual increase for *z* > 120 μm).

## Conclusions

The current research reports on the successful (UV-FRP)-assisted in-water site-specific photodeposition of PAA-GLP nanofilms on presilanized internal surfaces of fused silica microcapillaries (ID = 200 μm). The effectiveness of the silanization and photodeposition on the fused silica slides was determined by AFM and SEM–EDX spectroscopy. Confocal micro-Raman spectroscopy confirmed the photodeposition of polymer nanofilms on the inner surfaces of the microcapillaries.

## Future Prospective

In the future, the present results will allow the fabrication of a chemosensor or an adsorption device for GLP determination based on in-capillary-deposited nanofilms or nanoparticles of MIP-GLP. Moreover, the site-specific photodeposition will facilitate the manufacturing of an in-flow WGM optical microbubble resonator for GLP sensing. Thus far, studies on microfluidic sensors of GLP have demonstrated devising in-line (surface-enhanced Raman spectroscopy)-based sensor,^47^ electrophoresis-coupled disposable microchips with laser-induced fluorescence detection,^69^ an in-chamber copper-based water-gated organic field-effect transistor (WG-OFET),^70^ a (μ3D-paper-assisted) device functionalized with quantum dot-embedded poly(*N*-isopropylacrylamide) and *N*,*N*′-methylenebis(acrylamide) MIPs,^24^ and a methyl methacrylate MIP-based electrochemical lab-on-a-chip.^26^ Our procedure for on-surface photopolymerizing acrylamide encapsulating GLP enables simple and economical devising future systems for GLP detection and degradation.

Notably, we also demonstrated a robust (micro-Raman spectroscopy)-assisted imaging of PAA-GLP-coated microcapillary inner surfaces. Measuring the hydration level in the hydrogel and dried PAA-GLP enabled us to analyze the impact of GLP on PAA gelation. Moreover, the in-depth (*z*-stack) profiling confirmed the photodeposition of PAA-GLP on the internal surfaces. Our study demonstrates the usefulness of in-capillary micro-Raman spectroscopy imaging for GLP-related applied polymer science.

## Supporting Information Available

Silanization and confocal micro-Raman spectroscopy of the inner surface of a fused silica microcapillary; scanning electron microimages and energy-dispersive X-ray spectra of glyphosate-containing polyacrylamide thin films (PDF)

## List of Abbreviations

AA: acrylamide
ACN: acetonitrile
AIBN: azobis(2-methylpropionitrile), azobisisobutyronitrile
BAA: *N*,*N*′-methylenebis(acrylamide)
FRP: free-radical polymerization
GLP: glyphosate
MIP: molecularly imprinted polymer
PAA: polyacrylamide
PAA-GLP: glyphosate-containing polyacrylamide
TMOS: trimethoxy(octen-7-yl)silane
UV-FRP: (UV light)-activated free-radical polymerization
