# Supplementary material for: In-Capillary Photodeposition of Glyphosate-Containing Polyacrylamide Nanometer-Thick Films
Source: ACS Appl Polym Mater. 2022 Dec 1;5(1):223–35. doi: 10.1021/acsapm.2c01461 (PMC9841503; doi:10.1021/acsapm.2c01461)
Supplement: Supplementary file 1 — ap2c01461_si_001.pdf [file ap2c01461_si_001.pdf]

Supporting Information

for

In-capillary photodeposition of glyphosate-containing polyacrylamide nanometer-thick films

Jaroslaw Mazuryk* [1, 2], Katarzyna Klepacka [3, 6], Joanna Piechowska [3], Jakub Kalecki [3], Ladislav Derzsi [4], Piotr Piotrowski [5, 6], Piotr Paszke [5, 6], Dorota A. Pawlak [5, 6], Simone Berneschi [7], Wlodzimierz Kutner [1, 8], Piyush Sindhu Sharma [3]

[1] Electrode Processes Research Team, Institute of Physical Chemistry Polish Academy of Sciences, Kasprzaka 44/52, 01-224 Warsaw, Poland

[2] Bio & Soft Matter, Institute of Condensed Matter and Nanosciences, Université Catholique de Louvain, 1 Place Louis Pasteur, 1348 Louvain-La-Neuve, Belgium

[3] Functional Polymers Research Team, Institute of Physical Chemistry, Polish Academy of Sciences, Kasprzaka 44/52, 01-224, Warsaw, Poland

[4] Microfluidics and Complex Fluids Research Team, Institute of Physical Chemistry Polish Academy of Sciences, Kasprzaka 44/52, 01-224, Warsaw, Poland

[5] Faculty of Chemistry, University of Warsaw, Pasteura 1, 02-093, Warsaw, Poland

[6] ENSEMBLE3 sp. z o. o., Wólczyńska 133, 01-919, Warsaw, Poland

[7] Institute of Applied Physics "Nello Carrara" - National Research Council (IFAC-CNR), Via Madonna del Piano, 10, 50019, Sesto Fiorentino (FI), Italy

[8] Faculty of Mathematics and Natural Sciences. School of Sciences, Cardinal Stefan Wyszynski University in Warsaw, Wóycickiego 1/3, 01-938 Warsaw, Poland

Corresponding author: (*) Jaroslaw Mazuryk (JM), Institute of Physical Chemistry, Polish Academy of Sciences, Kasprzaka 44/52, 01-224 Warsaw, Poland; tel.: +48 22 343 2094, jmazuryk@ichf.edu.pl, ORCID account number: orcid.org/0000-0003-3311-7136; Current email address: jaroslaw.mazuryk@uclouvain.be; tel: +32 10 47 8460

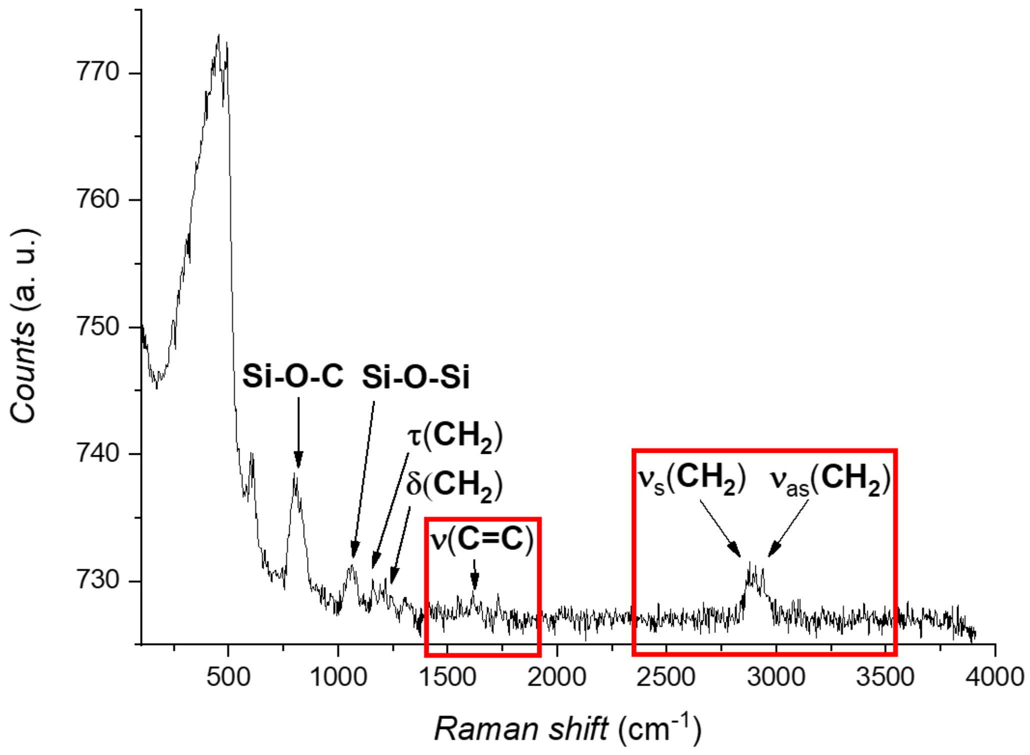

Figure S1. The Raman spectrum of TMOS deposited on the inner surface of the fused silica microcapillary from the 2 vol% TMOS anhydrous toluene solution for 2 h.

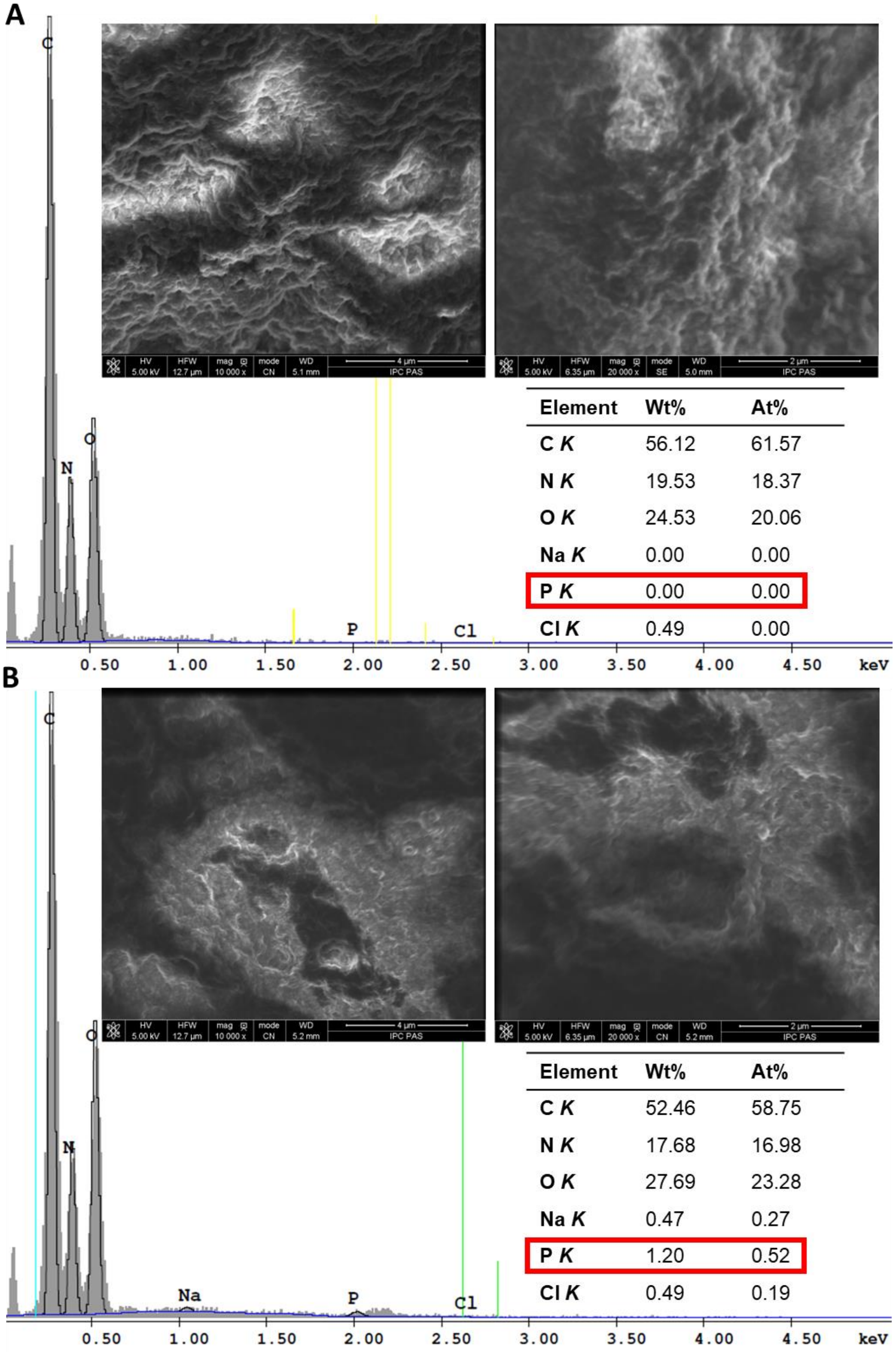

Figure S2. SEM microimages and EDX spectra of the (**A**) PAA and (**B**) PAA-GLP nanofilms drop-cast on gold plates. *K* – an electron shell.
